# INVESTIGATING ETHICAL DIMENSIONS AND TENSIONS WITH RESPECT TO THE USE OF RTLS IN DEMENTIA CARE

**DOI:** 10.1093/geroni/igae098.1441

**Published:** 2024-12-31

**Authors:** Alisa Grigorovich, Kelsey Harvey, Kyle Smilovsky, Josephine McMurray

**Affiliations:** Brock University, St. Catharines, Ontario, Canada; Brock University, St. Catharines, Ontario, Canada; Wilfrid Laurier University, Waterloo, Ontario, Canada; Wilfrid Laurier University, Waterloo, Ontario, Canada

## Abstract

Real-time location systems (RTLS) are increasingly being developed to track older adults with dementia across settings. Specifically, it is believed that such technologies can support automation of care tasks (e.g. locating) and the development of clinical algorithms to predict changes in health and wellbeing. The limited available research suggests that these technologies can have significant ethical implications, including increasing control over older adults, and undermining their rights. In this study, we explored the experiences and perceptions of RTLS by older residents, care partners, and organizational decision-makers in one care home in Ontario, Canada. Findings demonstrate that participants (N=47) had a limited understanding of RTLS (e.g., how it could be used, data storage, ethical issues) and this in turn influenced their perceptions of its value. Residents were generally unaware of its purpose and more concerned with its aesthetics. Care partners and organizational decision-makers valued the RTLS for enhancing their efforts to control risk to the physical safety of residents and believed this was central to enhancing quality of care. Most had limited awareness of residents’ preferences and believed that their cognitive impairment and/or frailty disqualified their current or prior values and wishes, including their desire for privacy and autonomy, and thus could be legitimately overridden in the interest of protecting their safety. We discuss these findings in relation to digital ageism and offer suggestions to guide future policy and educational interventions to better align development and use of RTLS and similar surveillance technologies with the wishes and preferences of older adults.

